# Multidimensional Predictors of Ranking-Based Competitive Success in National-Level Junior Tennis Players: Evidence for the Dominant Role of Physical Performance

**DOI:** 10.3390/sports14060253

**Published:** 2026-06-22

**Authors:** Rita Géczi, Gergely Géczi, László Tóth

**Affiliations:** 1 School of Doctoral Studies, Hungarian University of Sports Science, 1123 Budapest, Hungary; 2 Sport Economics and Decision Making Research Center, Hungarian University of Sports Science, 1123 Budapest, Hungary; 3 Department of Psychology and Sports Psychology, Hungarian University of Sports Science, 1123 Budapest, Hungary; 4 Teacher Training Institute, Hungarian University of Sports Science, 1123 Budapest, Hungary; 5 Institute of Health Promotion and Sport Sciences, Faculty of Education and Psychology, Eötvös Loránd University, 1117 Budapest, Hungary

**Keywords:** junior tennis, physical performance, executive functions, competitive anxiety, performance prediction, youth athletes, hierarchical regression

## Abstract

The aim of this study was to examine the relative contribution of physical performance, executive functions, and competitive anxiety to competitive success in junior tennis players. A total of 39 national-level junior athletes (20 males, 19 females) participated in the study. Physical performance was assessed using a standardized test battery including a 20 m sprint, standing long jump, agility test, and shuttle run. Executive functions were measured using the Adult Executive Functioning Inventory (ADEXI), while competitive anxiety was assessed with the Competitive State Anxiety Inventory-2 (CSAI-2). Competitive success was operationalized using ranking points. Hierarchical multiple regression analysis was conducted using log-transformed ranking points as the dependent variable. Age and sex explained 71.3% of the variance in LogRanking (R^2^ = 0.713, *p* < 0.001). The addition of physical performance variables provided a modest, non-significant increase in explained variance (ΔR^2^ = 0.068, *p* = 0.064). Executive functions (ΔR^2^ = 0.006, *p* = 0.645) and competitive anxiety (ΔR^2^ = 0.008, *p* = 0.801) did not provide additional explanatory power. In the final model, age and standing long jump were significant predictors of LogRanking. These findings suggest that ranking-based competitive success in junior tennis is strongly influenced by age-related and competition-exposure factors. Physical performance showed a limited additional contribution, while executive functions and competitive anxiety did not explain further variance in this sample.

## 1. Introduction

Performance in tennis is widely recognized as a multidimensional construct shaped by the interaction of physical, cognitive, and psychological factors. Contemporary models increasingly emphasize the need for integrative approaches that capture this complexity, rather than examining isolated domains [1,2]. Nevertheless, talent identification and monitoring systems in youth tennis continue to rely predominantly on physical performance indicators.

Physical capacities such as speed, agility, strength, and endurance are fundamental components of tennis performance and are routinely assessed within national development programs. These variables have been consistently associated with competitive success, particularly in junior populations. However, physical performance alone does not fully explain variability in competitive outcomes, suggesting that additional performance-relevant domains should be considered.

Among cognitive factors, executive functions (EF)—including working memory and inhibitory control—represent higher-order processes essential for behavioral regulation and goal-directed functioning. In sport contexts, particularly in open-skill disciplines such as tennis, these processes support rapid decision-making, attentional control, and adaptive responses to dynamic environments [3,4,5]. Athletes have been shown to outperform non-athletes in several EF domains, with advantages increasing alongside expertise level [3,4], although findings remain nuanced when distinguishing between hot and cool executive functions [6].

Executive functioning is influenced by both dispositional and contextual factors. Personality traits such as conscientiousness and neuroticism have been associated with working memory and inhibitory control [7,8,9], while lifestyle factors—including physical activity, sleep, sedentary behavior, and body composition—also contribute to EF variability [10]. From a developmental perspective, sport participation itself appears to enhance executive functioning, with meta-analytic evidence demonstrating improvements in working memory, inhibitory control, and cognitive flexibility among physically active youth [11]. At the same time, EF may be vulnerable under conditions of increased cognitive or neurological stress, such as following concussion [12].

In tennis specifically, executive functions have been linked to perceptual–cognitive processes, including visual attention, anticipation, and decision-making efficiency [12,13,14,15]. Moreover, executive control processes may play a critical role in maintaining performance under pressure, as working memory capacity has been shown to reduce anxiety-related performance decrements [10].

In parallel, competitive anxiety represents a key psychological factor influencing performance in tennis. Anxiety is conceptualized as a multidimensional construct comprising cognitive and somatic components, alongside self-confidence [16,17,18,19]. Elevated anxiety has been associated with impaired attentional control, reduced performance efficiency, and increased injury risk [20], particularly in high-pressure sport environments such as tennis [21].

Empirical findings indicate that anxiety responses are highly context-dependent. In youth tennis, anxiety levels vary as a function of age and gender, with competition contexts amplifying both cognitive and somatic components [22,23]. Real-time assessments have shown that cognitive anxiety increases from practice to competition settings [24], while psychophysiological studies reveal complex relationships between subjective anxiety and physiological responses such as heart rate variability [25]. Furthermore, situational pressure and prior performance errors have been shown to interact, increasing the likelihood of subsequent performance breakdowns [26], consistent with Attentional Control Theory [27,28].

Despite these findings, the anxiety–performance relationship remains inconsistent, with both debilitative and facilitative effects reported depending on individual and contextual factors [29]. In addition to anxiety, self-confidence has emerged as a robust predictor of sport performance, with meta-analytic evidence supporting its positive association with performance outcomes [30,31]. Other psychological characteristics, including fear of failure, perfectionism, and sensitivity to evaluation, have also been identified as contributors to anxiety in sport [32,33,34]. Broader contextual demands of tennis, such as travel, performance pressure, and social isolation, may further increase psychological stress [35].

Emerging evidence suggests that executive functions may play a regulatory role in the anxiety–performance relationship. Higher working memory capacity has been associated with improved performance and reduced susceptibility to anxiety-related disruption [36], while cognitive and psychological interventions—including attention control, imagery, and self-talk—have demonstrated effectiveness in improving both performance and anxiety regulation in tennis players [37,38,39]. In addition, data-driven approaches integrating psychological constructs with match analytics further highlight the importance of psychological processes in performance models [40].

Despite the growing body of literature examining physical, cognitive, and psychological factors, these domains are typically investigated in isolation. Few studies have examined their combined and incremental contributions within a single predictive model, particularly in junior tennis populations.

However, it remains unclear to what extent physical, cognitive, and psychological factors contribute independently and incrementally to competitive success when examined within an integrated framework in youth tennis.

To our knowledge, few studies have simultaneously examined the incremental contributions of physical, cognitive, and psychological variables within a single hierarchical model in junior tennis. Furthermore, the selection of variables in the present study was guided by both theoretical relevance to tennis performance and their applicability within a standardized national testing context.

Therefore, the aim of the present study was to investigate the relative contributions of physical performance, executive function, and competitive anxiety to competitive performance in junior tennis players using a hierarchical regression approach.

It was hypothesized that (1) physical performance would significantly predict competitive performance, (2) executive function would explain additional variance beyond physical factors, and (3) higher anxiety would be associated with poorer performance while self-confidence would be positively related.

## 2. Methods

### 2.1. Participants

A total of 39 junior tennis players (20 males, 19 females) participated in the study. All athletes were members of the Hungarian national talent development program and were actively competing with an official national ranking at the time of testing. The mean age of the participants was 13.718 ± 2.064 years.

The study protocol was approved by the Research Ethics Committee of the Hungarian University of Sports Science (approval number: MTSE-KEB/No21/2025). Written informed consent was obtained from all participants and their legal guardians prior to participation. All procedures were conducted in accordance with the Declaration of Helsinki.

Participant characteristics are presented in Table 1.

### 2.2. Study Design

A cross-sectional study design was applied. All measurements were conducted within the same testing period under standardized conditions as part of the annual performance evaluation protocol of the Hungarian Tennis Federation. Physical and psychological assessments were performed in close temporal proximity to ensure consistency across variables.

## 3. Measures

All variables were selected based on their theoretical relevance to tennis performance, previous empirical evidence, and their feasibility within the standardized assessment protocol of the Hungarian Tennis Federation.

### 3.1. Performance Outcome

Competitive success was operationalized using official ranking points obtained from the Hungarian Tennis Federation.

Ranking points reflect competitive results accumulated within a rolling one-year period. However, they may also be influenced by tournament participation frequency, competition level, and access to higher-category events. Therefore, ranking points should be interpreted as a proxy of competitive success rather than a direct measure of performance ability.

### 3.2. Physical Performance Tests

Participants completed a standardized physical test battery routinely used in the national talent identification program. The following variables were included in the analysis:

• Speed: 20 m sprint time;
• Lower-body explosive power: standing long jump;
• Agility: change-of-direction test;
• Aerobic capacity: shuttle run test.

These variables were selected based on their established relevance in national talent identification protocols and their demonstrated association with tennis-specific performance demands.

### 3.3. Executive Functions

Executive functions were assessed using the Adult Executive Functioning Inventory (ADEXI) [41]. The questionnaire provides two subscales:

• Working memory (ADEXI-MM);
• Inhibitory control (ADEXI-GAT).

Higher scores indicate greater executive function difficulties.

The ADEXI captures ecologically relevant aspects of executive functioning in everyday behavior, which may be particularly meaningful in applied sport settings. The use of ADEXI was also justified by the need to apply a consistent measurement tool across a wide age range, as child-specific instruments such as the CHEXI are typically validated only up to early adolescence. Given the heterogeneous age distribution of the sample, ADEXI provided a unified assessment framework across participants.

This approach allowed for comparability across age groups, although it may have reduced sensitivity to developmental differences.

### 3.4. Competitive Anxiety

Competitive anxiety was measured using the Competitive State Anxiety Inventory-2 (CSAI-2) [16], which includes three subscales:

• Cognitive anxiety;
• Somatic anxiety;
• Self-confidence.

The CSAI-2 is widely used in sport psychology research to assess athletes’ psychological states related to competition.

Given the age of the participants, the researcher was present during questionnaire administration to assist with comprehension when needed. Clarifications were provided upon request to ensure that participants accurately understood the items, while care was taken not to influence their responses.

Participants were instructed to complete the CSAI-2 retrospectively by recalling how they typically felt immediately before competing at the European Championships. This approach was chosen to standardize the competitive context across participants and to capture anxiety responses associated with a highly meaningful competition.

### 3.5. Statistical Analysis

All statistical analyses were performed using JASP software (version 0.19.3.0). Descriptive statistics (mean ± standard deviation) were calculated for all variables.

Hierarchical multiple linear regression analysis was conducted to examine the relative contribution of different variable domains to competitive success (ranking points). Because ranking points showed substantial positive skewness and violated normality assumptions, a logarithmic transformation was applied prior to regression analyses. The transformed variable (LogRanking) was used as the dependent variable in all regression models.

• Step 1: age and sex (control variables);
• Step 2: physical performance variables (20 m sprint, standing long jump, agility, shuttle run);
• Step 3: executive function variables (ADEXI-MM, ADEXI-GAT);
• Step 4: psychological variables (CSAI-2 cognitive anxiety, somatic anxiety, self-confidence).

Changes in explained variance (ΔR^2^) were used to evaluate the incremental contribution of each block.

Statistical significance was set at *p* < 0.05.

Multicollinearity diagnostics indicated acceptable to moderate VIF values, with the highest value observed for standing long jump (VIF = 5.21).

Regression diagnostics indicated no major violations of normality, homoscedasticity, or multicollinearity assumptions.

In addition to *p*-values, effect sizes (R^2^ and ΔR^2^) were examined to provide a more comprehensive interpretation of the practical significance of the findings.

Descriptive statistics for all study variables are presented in Table 2. Because sex was included as a control variable in all regression models, the reported associations represent effects beyond sex-related differences. Furthermore, sex did not emerge as a significant predictor in any model.

## 4. Results

### 4.1. Hierarchical Linear Regression Analysis

A hierarchical linear regression analysis was conducted to examine the extent to which demographic, physical, cognitive, and psychological variables predicted ranking-based competitive success, operationalized as log-transformed ranking points (LogRanking).

In the first step, age and sex were entered as control variables. This model was significant, F(2,36) = 44.70, *p* < 0.001, explaining 71.3% of the variance in LogRanking (R^2^ = 0.713).

In the second step, physical performance variables (20 m sprint, standing long jump, agility, and shuttle run) were added. This resulted in an additional 6.8% of explained variance (ΔR^2^ = 0.068), although the improvement did not reach statistical significance (*p* = 0.064). The overall model explained 78.1% of the variance (R^2^ = 0.781).

The inclusion of executive function variables produced only a marginal increase in explained variance (ΔR^2^ = 0.006, *p* = 0.645).

Likewise, competitive anxiety variables did not significantly improve the model (ΔR^2^ = 0.008, *p* = 0.801).

Detailed results of the hierarchical regression analysis are presented in Table 3.

**Table 3:** Summary of hierarchical regression models predicting ranking points across variable blocks.

Model	R^2^	Adjusted R^2^	ΔR^2^	F Change *p*
M1	0.713	0.697	0.713	<0.001
M2	0.781	0.739	0.068	0.064
M3	0.787	0.730	0.006	0.645
M4	0.795	0.711	0.008	0.801

### 4.2. Final Model Predictors

In the final model, age remained the strongest predictor of LogRanking (β = 0.962, *p* < 0.001). Standing long jump was also retained as a statistically significant predictor (β = −0.443, *p* = 0.035), although its negative coefficient should be interpreted cautiously due to the strong association between age and physical development within the sample. None of the executive function or competitive anxiety variables significantly predicted LogRanking.

Detailed regression coefficients of the final hierarchical model are presented in Table 4.

Overall, the results indicate that age accounted for the largest proportion of explained variance in LogRanking, while physical variables provided a modest additional contribution. Cognitive and psychological variables contributed minimally to the final model.

## 5. Discussion

### 5.1. Main Findings

The present study examined the relative contribution of demographic, physical, cognitive, and psychological variables to ranking-based competitive success in junior tennis players. The results showed that age was the strongest predictor of ranking outcomes. Physical performance variables provided a modest additional contribution to explained variance, whereas executive functions and competitive anxiety did not explain additional variance beyond demographic and physical factors.

Importantly, the present findings should be interpreted in relation to ranking-based outcomes rather than as direct indicators of pure performance ability, as ranking points in junior tennis also reflect competition exposure and access to competitive opportunities.

Among the physical measures, standing long jump emerged as the only statistically significant physical predictor in the final model. However, this finding should be interpreted with caution, as standing long jump showed a positive bivariate association with LogRanking, whereas the regression coefficient became negative after controlling for age and the other physical variables. This apparent inconsistency likely reflects shared variance among age, physical development, and performance characteristics within the sample, suggesting a suppression effect rather than a true negative relationship between lower-body power and competitive success. The positive bivariate correlation between standing long jump and LogRanking (r = 0.423, *p* = 0.007, 95% CI [0.124, 0.652]) further supports this interpretation.

Notably, age alone explained a substantial proportion of variance in ranking outcomes, highlighting the strong influence of developmental and competitive exposure factors in junior tennis. This finding likely reflects both developmental differences and age-related variation in competition exposure within national-level junior tennis.

### 5.2. Interpretation of Physical Dominance

The observed importance of lower-body explosive power is consistent with the specific physiological and movement demands of tennis. High-level tennis performance requires rapid acceleration, repeated changes in direction, and explosive stroke execution, all of which are strongly dependent on neuromuscular performance.

These findings align with previous research highlighting lower-body power and neuromuscular performance as important determinants of success in junior tennis populations. Similar findings were reported by Durmuş (2025) [2], whose multidimensional analysis of junior tennis players demonstrated significant relationships between ranking position and multiple physical performance indicators.

This reinforces the idea that physical capacities may contribute to ranking-based competitive success during developmental stages; however, their incremental contribution beyond age and sex did not reach statistical significance in the present model.

### 5.3. Why Psychological Variables Were Not Significant

Contrary to the initial hypotheses, neither executive functions nor competitive anxiety showed a direct relationship with ranking performance. Importantly, the absence of statistical significance should not be interpreted as evidence of irrelevance. Rather, psychological and cognitive factors may exert indirect, context-dependent, or developmental effects that are not fully captured by cumulative ranking outcomes within a cross-sectional design.

Several explanations may account for this finding.

First, ranking points represent a cumulative performance indicator influenced by competition exposure, tournament selection, and participation frequency. As such, ranking may not accurately reflect momentary performance states, where psychological factors are more likely to exert their effects.

Second, psychological variables may influence performance indirectly rather than directly. For example, executive functions may contribute to training quality, decision-making, and consistency, while anxiety regulation may affect performance under specific competitive conditions rather than across aggregated outcomes.

Third, in junior populations, cognitive and emotional regulation processes are still developing. This developmental variability may reduce the observable impact of psychological factors in cross-sectional designs.

Finally, it is possible that the instruments used capture general tendencies rather than competition-specific psychological states, which may further attenuate their relationship with ranking-based outcomes.

### 5.4. Integration with Theoretical Models

The findings partially support multidimensional models of sport performance but also highlight an important nuance: not all performance domains contribute equally to all outcome measures.

While theoretical frameworks emphasize the interaction between physical, cognitive, and psychological factors, the present results suggest that performance indicators such as ranking points may be predominantly sensitive to physical capacities.

Consequently, the present findings should not be interpreted as evidence that psychological factors are unimportant in junior tennis but rather that their influence may not be adequately captured by ranking-based outcome measures within a cross-sectional framework.

### 5.5. Practical Implications

From an applied perspective, the findings highlight the importance of physical development, particularly lower-body power, in junior tennis. However, ranking-based outcomes should be interpreted cautiously, as they may reflect competition exposure in addition to performance ability. Psychological factors remain important for athlete development, although their influence may not be directly captured by cumulative ranking indicators.

## 6. Limitations

Several limitations of the present study should be acknowledged.

First, the use of ranking points as an indicator of competitive success may not fully reflect underlying performance ability. Ranking points are influenced not only by match outcomes but also by competition exposure, including the number of tournaments played. As a result, older players may have greater competition exposure and may participate in a larger number of tournaments, including higher-level events, which could contribute to higher ranking point totals.

Second, the relatively small sample size (N = 39) limits the statistical power of the analyses, particularly in detecting smaller effects of psychological variables. Although the sample size was relatively small, it reflects the structure of a national-level junior cohort, thereby enhancing the ecological validity of the findings despite limited statistical power. Given the relatively large number of predictors relative to the sample size, the regression models may have been underpowered to detect smaller effects, particularly for psychological variables. Therefore, non-significant findings should be interpreted cautiously.

Third, the cross-sectional design restricts the ability to draw causal inferences. While physical performance variables were found to be strong predictors of ranking, it cannot be determined whether these capacities directly lead to improved performance or whether higher-performing players develop superior physical qualities over time.

Fourth, psychological variables (executive functions and competitive anxiety) were assessed at a single time point, which may not capture their dynamic and context-dependent nature. In competitive sports, psychological states can fluctuate considerably depending on situational factors such as match importance, opponent level, or environmental stressors.

Finally, the study did not account for additional factors that may influence tennis performance, such as technical-tactical skills, coaching quality, injury history, or training load. These variables may interact with both physical and psychological characteristics and contribute to competitive success.

Another limitation concerns the assessment of executive functions using a self-report measure (ADEXI). While the questionnaire captures ecologically relevant aspects of everyday cognitive functioning, self-reported data may be subject to bias, particularly in younger populations. Future studies may benefit from incorporating objective cognitive assessments (e.g., reaction time tasks, inhibitory control paradigms), which could provide more sensitive and performance-related indicators of executive functioning in sport contexts. Nevertheless, the ADEXI was selected because it allowed the use of a single instrument across the entire age range of the sample (11–18 years), thereby ensuring methodological consistency across participants.

Furthermore, the relatively low participant-to-predictor ratio may have affected model stability and reduced the likelihood of detecting smaller effects.

Important determinants of tennis performance, including technical, tactical, training-history, and injury-related variables, were not available in the present dataset. Consequently, the reported models should be interpreted as representing only a partial explanation of competitive success.

### Future Directions

Future research should aim to include larger and more diverse samples and adopt longitudinal designs to better capture developmental and performance-related changes over time. In addition, incorporating objective cognitive assessments and more sensitive, context-specific performance indicators—particularly those reflecting performance under pressure or in critical match situations—may provide a more comprehensive understanding of the role of psychological factors in tennis. Furthermore, examining the interaction between physical and psychological variables may offer deeper insight into the complex determinants of performance in junior tennis.

## 7. Conclusions

The present study suggests that physical performance variables, particularly lower-body explosive power, showed meaningful associations with ranking-based competitive success, although their incremental contribution beyond demographic factors did not reach statistical significance.

While executive functions and competitive anxiety were theoretically relevant, they did not contribute additional explanatory power beyond physical and demographic factors in the present model. This suggests that performance models in youth tennis may be predominantly driven by physical capacities when assessed using ranking-based outcomes.

Importantly, these findings do not diminish the role of psychological factors but rather indicate that their influence may be indirect, developmental, or context-dependent. Future research should therefore adopt more sensitive and context-specific performance measures to better capture the complex interaction between physical and psychological determinants of performance.

## Figures and Tables

**Table 1:** Participant characteristics.

Variable	Value
Age (years), mean ± SD	13.72 ± 2.06
Age range (years)	11–18
Males, *n* (%)	20 (51.3%)
Females, *n* (%)	19 (48.7%)
11 years, *n* (%)	6 (15.4%)
12 years, *n* (%)	7 (17.9%)
13 years, *n* (%)	7 (17.9%)
14 years, *n* (%)	7 (17.9%)
15 years, *n* (%)	3 (7.7%)
16 years, *n* (%)	3 (7.7%)
17 years, *n* (%)	5 (12.8%)
18 years, *n* (%)	1 (2.6%)

**Table 2:** Descriptive statistics of demographic, physical, cognitive, and psychological variables.

Variable	Mean ± SD	Min	Max
Age (years)	13.7 ± 2.1	11	18
Sprint 20 m (s)	3.40 ± 0.26	2.82	3.90
Standing long jump (cm)	200.3 ± 27.5	156	286
Agility (s)	6.25 ± 0.56	5.35	7.86
Shuttle run	79.4 ± 23.3	50	151
ADEXI-MM	1.84 ± 0.41	1.11	2.78
ADEXI-GAT	2.52 ± 0.65	1.40	4.40
CSAI cognitive	1.75 ± 0.54	1.00	3.17
CSAI somatic	1.54 ± 0.44	1.00	2.83
CSAI confidence	2.76 ± 0.55	1.33	3.67
Ranking points	2615.1 ± 3655.6	260	18,350

**Table 4:** Final model coefficients.

Predictor	B	SE	β	95% CI for B	*p*	VIF
Age	0.499	0.074	0.962	[0.347, 0.651]	<0.001	2.694
Sprint 20 m	−1.234	0.760	−0.299	[−2.793, 0.326]	0.116	4.453
Standing long jump	−0.017	0.008	−0.443	[−0.033, −0.001]	0.035	5.210
Agility	−0.211	0.312	−0.110	[−0.851, 0.430]	0.505	3.477
Shuttle run	−0.006	0.007	−0.137	[−0.021, 0.009]	0.395	3.304
ADEXI-GAT	0.112	0.197	0.068	[−0.292, 0.517]	0.573	1.857
ADEXI-MM	−0.255	0.304	−0.098	[−0.879, 0.369]	0.409	1.807
Sex	0.071	0.242	—	[−0.426, 0.569]	0.771	1.729
CSAI cognitive	0.096	0.233	0.049	[−0.382, 0.575]	0.683	1.841
CSAI somatic	−0.223	0.280	−0.092	[−0.797, 0.351]	0.432	1.747
CSAI confidence	−0.106	0.211	−0.054	[−0.539, 0.327]	0.619	1.528

Note. Dependent variable = LogRanking. B = unstandardized coefficient; β = standardized coefficient; CI = confidence interval; VIF = variance inflation factor.

## Data Availability

The data presented in this study are available from the corresponding author upon reasonable request.
